# Comorbidity in patients with diabetes mellitus: impact on medical health care utilization

**DOI:** 10.1186/1472-6963-6-84

**Published:** 2006-07-04

**Authors:** Jeroen N Struijs, Caroline A Baan, Francois G Schellevis, Gert P Westert, Geertrudis AM van den Bos

**Affiliations:** 1 Department for Prevention and Health Services Research, National Institute of Public Health and the Environment, A.van Leeuwenhoekstraat 9, 3720 BA Bilthoven, The Netherlands; 2 NIVEL, Netherlands Institute for Health Services Research, Otterstraat 118 – 124 3513 CR Utrecht, The Netherlands; 3 Department of Social Medicine, Academic Medical Centre, University of Amsterdam, Meibergdreef 9, 1105 AZ Amsterdam, The Netherlands

## Abstract

**Background:**

Comorbidity has been shown to intensify health care utilization and to increase medical care costs for patients with diabetes. However, most studies have been focused on one health care service, mainly hospital care, or limited their analyses to one additional comorbid disease, or the data were based on self-reported questionnaires instead of health care registration data. The purpose of this study is to estimate the effects a broad spectrum of of comorbidities on the type and volume of medical health care utilization of patients with diabetes.

**Methods:**

By linking general practice and hospital based registrations in the Netherlands, data on comorbidity and health care utilization of patients with diabetes (n = 7,499) were obtained. Comorbidity was defined as diabetes-related comorbiiabetes-related comorbidity. Multilevel regression analyses were applied to estimate the effects of comorbidity on health care utilization.

**Results:**

Our results show that both diabetes-related and non diabetes-related comorbidity increase the use of medical care substantially in patients with diabetes. Having both diabeterelated and non diabetes-related comorbidity incrases the demand for health care even more. Differences in health care utilization patterns were observed between the comorbidities.

**Conclusion:**

Non diabetes-related comorbidity increases the health care demand as much as diabetes-related comorbidity. Current single-disease approach of integrated diabetes care should be extended with additional care modules, which must be generic and include multiple diseases in order to meet the complex health care demands of patients with diabetes in the future.

## Background

Comorbidity, defined as the occurrence of one or more chronic conditions in the same person with an index-disease, occurs frequently among patients with diabetes [1,2]. Currently, integrated diabetes care programs focus on diabetes-related comorbidities like cardiovascular diseases, retinopathy, nephropathy and diabetic foot. However, patients with diabetes do not only have diabetes-related comorbidity but also have non diabetes-related comorbidity, such as depression and musculoskeletal diseases [2-5]. With the ongoing population aging of Western societies, not only the number of patients with diabetes is expected to increase, but also the number of patients with diabetes with comorbidity. This implies that the current single disease management approach is not applicable to a large part of the patients with diabetes in the future.

Comorbidity among patients with diabetes is associated with considerable consequences for health care and related costs [6-15]. Comorbidity has been shown to intensify health care utilization and to increase medical care costs for patients with diabetes. However, most studies have been focused on one health care service, mainly hospital care [6,11,12], or limited their analyses to one additional comorbid disease [7,11]. In addition, previous studies on multidisciplinary health care utilization were based on self-reported questionnaires instead of health care registration data [9]. We elaborated on these studies by taking into account a broad spectrum of comorbidities and focusing on multiple health care services by linking data of different health care registrations.

The aim of this study is to examine the impact of comorbidity in patients with diabetes on the use of general practitioner (GP) care, medical specialist care and hospital care. After presenting the prevalence figures of comorbidity in patients with diabetes, the following questions will be addressed: what is the effect of comorbidity on the type and volume of health care utilization of patients with diabetes? And, which comorbidity pattern has the highest additional effect?

A better understanding of the effects of comorbidity on the type and volume of medical health care utilization is essential to gain insight into future health care demands of patients with diabetes.

## Methods

### Design

Data on comorbidity and health care utilization were derived from three health care registrations. Firstly, data were obtained from the second Dutch National Survey of General Practice (DNSGP-2)[16]. The DNSGP-2 was carried out in 104 general practices (including 195 General Practitioners (GPs)) in the Netherlands in 2001. The DNSGP-2 includes approximately 385,000 patients, with data about 1,5 million encounters, 2,2 million prescriptions of pharmaceuticals and 170,000 first time referrals to other health care providers. Diagnoses were coded according to the International Classification of Primary Care (ICPC) [17]. The study population is representative of the Dutch population regarding age, gender and type of health care insurance [17]. Dutch GPs are the gatekeepers for medical specialised care and nearly all non-institutionalised patients are listed to a GP.

Secondly, information about consultations of medical specialists were obtained from the National Register of ambulatory care for the years 1999 until 2002 [18]. In the Netherlands, medical specialists work in hospitals. In 2001, 108 out of the 120 hospitals in the Netherlands have joined the National Register of ambulatory care. Forty-five percent of the hospitals register all visits to a medical specialist. The other hospitals register only the first visit during a certain calendar year. Records comprise date of birth, gender, area zip code, medical specialty and date of consultation. In 2001, about 8,7 million consultations of medical specialists were registered in this register.

Thirdly, data were obtained from the National Medical Register for the years 1999 until 2002 [19]. This database has an almost complete coverage (99%) of all hospital admissions in the Netherlands. Records comprise date of birth, gender, area zip code, length of stay, diagnosis at discharge and vital status at discharge. Diagnoses at discharge are coded according to the International Classification of Diseases, Ninth Revision [20]. The total number of hospital admissions amounted to about 1,5 million in 2001.

### Patient linkage

We used gender, date of birth and area zip code of the patient as linking variables for the linkage of records in the different registrations, since a unique identifier is not available in the Netherlands [21]. A pilot linkage was successfully carried out in order to evaluate the representativeness of the linkage and the resulting population [22]. The starting point of the linkage is a GP referral. On the basis of each GP referral we searched for corresponding records in the ambulatory care and hospital registers. We restricted the period in the registers for each separate referral to 180 days before and 360 days after the referral date. Linkages outside this period were excluded from the analyses.

### Study population

First, patients with missing values on any of the linking variables and patients with a non-unique combination of linking variables in the DNSGP-2 were excluded from the analyses (n = 24,193, 6%). Subsequently, we selected patients who had contacted their GP for diabetes mellitus (ICPC code T90) in 2001 (n = 9,313). In addition, we excluded patients with missing information on socioeconomic status, which was indicated by highest educational level (n = 1,814). Socioeconomic status was included as a confounding variable. In total, 7,499 diabetes mellitus patients were included in the analyses.

### Measures

We included the following measures of medical health care utilization in the analysis: the number of contacts with the GP (per year), total number of prescriptions by the GP (per year), mean number of prescriptions per GP contact, consultation of the medical specialist (yes/no), the number of consultations of the medical specialist (per year) (of those who consulted the medical specialist), hospital admission (yes/no), the number of hospital admissions (per year) (of those who were admitted to the hospital), the average length of stay per hospital admission, and the total number of hospital days (per year).

Micro- and macrovascular comorbidity was defined as chronic conditions which have a vascular relationship with diabetes or which can be seen as vascular complications of (the treatment) of diabetes. Non-vascular comorbidity was defined as chronic conditions of which the relationship with diabetes is unrelated or still not understood [23], but are not the result of vascular damage. Comorbid conditions were based on diagnosis recorded by GPs. Diabetes-related comorbidity included the following chronic conditions: heart diseases (K74–K77), stroke (K90), retinopathy (F83), nephropathy (U99) and diabetic foot (S97). The non diabetes-related comorbidity was defined as depression (P76), lung diseases (Chronic Obstructive Pulmonary Disease (COPD) (R91, R95) and asthma (R96)), musculoskeletal diseases, neurological diseases and cancer. Musculoskeletal disease was defined as any of the following conditions: low back pain (L02, L03 and L86), shoulder and neck pain (L01, L08) and osteoarthritis of knee (L13), hip (L15) or spine (L84, L89–91). Neurological disease was defined as multiple sclerosis (N86), Parkinson's disease (N87) and epilepsy (N88). Cancer was defined as non-Hodgkin disease (B74), stomach cancer (D74), colon cancer (D75), oesophagus cancer (D77), lung cancer (R84), skin cancer (S77), breast cancer (X76), and prostate cancer (Y77).

### Statistical analyses

We categorized comorbidity in two ways: 1) the presence/absence of any comorbidity, and 2) four mutually exclusive categories of no comorbidity, vascular comorbidity only, non-vascular comorbidity only, and both vascular and non-vascular comorbidity.

First, we compared the patient characteristics and assessed the differences in health care utilization between the patient groups by performing chi-square tests and t-tests (table 1).

Subsequently, multilevel analyses were applied to estimate the effects of comorbidity on health care utilization. Multilevel analyses were used because of the two-level structure of the data (i.e. practice level and patient level), allowing us to adjust for variation among GP-practices (e.g. variation regarding diabetes control and prescription behaviour) (table 2 and 3). We estimated the effect of comorbidity on health care utilization in three ways: 1) the effect of the presence of any comorbidity (table 2), 2) the effect of the type of comorbidity (vascular or non-vascular) (table 2), and 3) the effect of specific comorbidity (table 3).

The presence of any comorbidity, type of comorbidity and specific comorbidities were coded as dummy variables. In all multilevel analyses, we adjusted for age, gender and educational level of the patient (all fixed effects) and GP-practices (random effect) and the patient group without comorbidity was used as reference group. In all multilevel analyses, variances in the health care utilization were assessed both at the GP practice-level and the individual level. The size of the GP practice level variance (between GP-practice variation) and the size of the individual-level variance are expressed relative to the overall variance. The effect sizes as results of the multilevel linear and logistic regression analyses were expressed as coefficient (β) with 95% confidence intervals (CI) or as odds ratio (OR) with the corresponding 95% CI.

## Results

Of all patients referred by their GP to a medical specialist 87% could be linked to a hospital admission, an outpatient treatment or an outpatient visit.

In table 1, data on patient characteristics and health care utilization measures are listed. About 44% (n = 3,324) of the patients with diabetes had any additional comorbidity, while 56% of the patients with diabetes had no additional comorbidity (n = 4,175). The comorbid patients were categorized into one, two and three or more comorbidities. Patients with diabetes without comorbidity were younger (62.6 versus 66.9, 71.1 and 72.9 years of age), were more likely to be male (48.4% versus 43.7%, 40.4% and 41.2% males) and higher educated (10.5% versus 7.4%, 3.6% and 5.3% high educated patients). After specifying the comorbidity as vascular or non-vascular, significant differences in patient characteristics between the comorbid patient groups were observed. Patients with vascular comorbidities were more likely to be male compared with the other comorbid patient groups (51.2% males versus 39.0% and 42.5%, respectively). Patients with both vascular and non-vascular comorbidity were on average older compared with patients with either vascular or non-vascular comorbidity (73.1 versus 71.5 and 64.9 years of age respectively) and more likely to be lower educated (4.0% versus 6.5% and 7.3% high educated patients respectively).

### Medical health care utilization

A clear gradient was observed between the number of comorbidities and the increase of health care utilization, with the exception of the percentage of patients with prescriptions, which was in all patient groups almost 100% (table 1). No large differences were observed in the use of GP care between patients with either vascular comorbidity or non-vascular comorbidity. Patients with both types of comorbidity visit their GP more frequently than patients with either vascular comorbidity or non-vascular comorbidity (23.2 vs. 15.0 and 14.3 GP contacts respectively). The same pattern was observed for the total number of prescriptions per year.

Fewer patients with diabetes only consulted the medical specialist (12.3%) compared to the patient groups with vascular comorbidity, non-vascular comorbidity or both (17.8%, 26.5% and 33.3% respectively). The same pattern was observed for the mean number of consultations with the medical specialist, for being admitted to the hospital, the mean number of hospital admissions and for the total number of hospital days. The average length of stay in the hospital for patients with non diabetes-related comorbidity was relatively shortest (5.8 days).

Multilevel modeling showed that 16% of the total variance in the number of GP contacts was related to the GP practice level, and that 84% occurred at the individual level (not tabulated). This means that the number of GP contacts of patients with diabetes within GP-practices are correlated, and as a consequence, that using one-level linear regression models will lead to less valid estimates. Variance at the GP practice level was also substantial in the number of GP prescriptions (11%) and having consulted the medical specialist (8%) but diminished in other health care utilization measures, varying from 5% (number of consultations with the medical specialist) till 1% (average length of stay).

In the multilevel multivariate analyses, a strong gradient was observed between the number of comorbidities and health care utilization measures after adjustment for age, gender, educational level and GP-practice (table 2). An increasing number of comorbidities resulted in an increase in health care utilization for all measures.

For patients with vascular comorbidity or non-vascular comorbidity, no differences were found for the number of GP visits (4.6 and 4.7 respectively), number of hospital admissions (0.3 and 0.3 respectively) and average length of stay in the hospital (0.7 days and 0.6 days respectively). Patients with both vascular and non-vascular comorbidity have the highest health care utilization with respect to both GP care, medical specialist care and hospital care.

Table 3 shows that each specific comorbidity increased the use of GP care substantially, especially diabetic foot (8.5 additional GP contacts and 20.9 additional prescriptions (mainly bandages)), compared with patients with diabetes without comorbidity.

The effects of comorbidity on the use of medical specialist care differ between the specific comorbidities. For retinopathy, nephropathy, diabetic foot and neurological diseases no significant differences were observed in the number of consultations with the medical specialist compared with patients with diabetes without comorbidity. In addition, the ORs for having consulted the medical specialist differed significantly only for heart diseases, stroke and musculoskeletal diseases as compared with the reference group (OR = 1.7, OR = 2.0 and OR = 3.6 respectively).

Patients with diabetes, having a heart disease or stroke as a comorbid disease, showed the relatively largest increase in hospital care (3.1 and 3.3 additional hospital days respectively). The average length of stay was significantly higher in most of the comorbidities, with the exception of retinopathy, nephropathy and neurological diseases. For heart diseases, stroke, musculoskeletal diseases, medical health care utilization was significant increase compared with the reference group for all health care measures. The other comorbid diseases resulted in a significant increase of health care utilization for only two or three health care utilization measures.

## Discussion

Not surprisingly, a strong correlation was observed in this study between the number of comorbidities and the use of GP care, ambulatory specialist care and hospital admissions. However, we found no systematic differences between patients with either vascular or non-vascular comorbidity. Patients with both vascular and non-vascular comorbidity showed the highest health care utilization pattern. This is mainly caused by the higher number of comorbidities in this patient group.

Our results demonstrated that patients without comorbidity use little care. The large impact on health care utilization of patients with diabetes occurs when diabetes is included in a constellation of vascular and non-vascular comorbidities. Therefore, health care providers should routinely monitor patients with diabetes with respect to comorbidity. Case-finding protocols as mentioned by Gijsen et al. [8], should be developed and implemented in integrated diabetes care programs.

Moreover, our results demonstrated that non-vascular comorbidities are as important utilization drivers as vascular comorbidities. In addition to the beneficial effects of it on the quality of life of not having any comorbidity [8], prevention of comorbidity can possibly also curb the growing demands for health care. Until now, prevention in integrated diabetes care programs focuses mainly on micro- and macrovascular comorbidity. Our study shows that this focus is too limited, since additional non-vascular comorbidities in patients with diabetes increase the health care utilization as much as vascular comorbidities.

Different comorbid conditions have different effects on health care utilization. Diabetic foot results in a large increase in the use of GP care, but not in the use of medical specialist care and hospital care. Coronary heart diseases, stroke, depression, musculoskeletal diseases and cancer result in a substantially increase in both GP care, medical specialist care and hospital care. Our finding that the average length of stay in the hospital increases in most comorbidities, is in accordance with findings from other studies [24,25].

Limitations of the study need to be considered in interpreting the results. Firstly, we obtained data about health care utilization by medical record linkage of different registrations, which introduced some selection bias. Linkage probabilities between older and younger patients differ because of different rates of changes of address [26]. Older people move less often than younger people and therefore have higher linkage probabilities since area zip code is one of the linkage variables. Therefore the hospital utilization of the younger patients, i.e. the patient group with diabetes only, could be underestimated.

Secondly, the number of consultations by the medical specialist in the linkage is an underestimation of the actual number of consultations, since only 45% of the hospitals in the National Ambulant Register register all consultations. Since this inaccuracy in the registration of consultations to the medical specialist applies for all patient groups, it is not likely that this has biased our results.

Thirdly, for a large number of patients information on educational level was missing and they were therefore excluded from the analyses. Patients with missing data on educational level might differ from the included patients, which may have influenced our results. However, additional analyses showed that the health care utilization of patients with unknown educational level did not differ from the patients with known educational level.

Fourth, the prevalence's of retinopathy and nephropathy are substantially lower than observed in another study [27]. A reason for this underregistration could be that retinopathy and nephropathy are not registered adequately in the GP records, since these vascular comorbidities are often treated and registered in integrated care programs and resulting in suboptimal registration in the GP records. This underestimation of the number of patients with retinopathy and nephropathy might lead to an underestimation of the effects of these vascualar comorbidities on GP care, medical specialist care and hospital care.

In our analyses, we focused on medical care only. Future research should also include long-term care, e.g. home care, nursing home care and rehabilitation care, in order to fully understand the effects of comorbidity.

## Conclusion

Non-vascular comorbidities are as important utilization drivers as vascular comorbidity for patients with diabetes, while patients without comorbidity use little care. These results underline the importance of primary and secondary prevention of all comorbidity in patients with diabetes.

Our results demonstrated also that the number of comorbidities is a strong predictor for the volume of medical health care utilization and support the increasing interest in specific patterns of comorbidity in clinical practice [9,28]. Together with the ongoing aging of the Dutch population and the increase in the number of patients with comorbidity, this may have important implications for integrated diabetes care programs in the Netherlands. From a public health perspective, the sum score of the number of comorbidities is a strong tool in predicting the future health care utilization of patients with diabetes.

Current single disease management approach is not suitable for a large part of the patients with diabetes. Current diabetes care programs must be extended to include additional care modules, which must be generic and include multiple chronic diseases. Perhaps in the long term, current diabetes care programs must be integrated with other chronic diseases care programs. The results of the first trials in this area are promising [29], and these integrated chronic care programs will be more equipped to meet the complex health care demands of patients with diabetes and to reduce the existing gap between needs of patients with diabetes and the supply of diabetes care in the future. From a public health perspective, the sum score of the number of comorbidities in patients with diabetes appears to be a strong predictor in estimating future health care demands.

## Competing interests

The author(s) declare that they have no competing interests.

## Authors' contributions

JS and CAB iniated the idea of the manuscript, performed the statistical analyses (JS) and wrote the manuscript. FGS and GPW were responsible for the acquisition of the data and the interpretation of the data and analyses. They reviewed all drafts of the manuscript.

GAMB participated in the design of the study, contributed and reviewed all drafts of the manuscript and supervised the whole study. All authors have read and approved the last version of the manuscript.

## Pre-publication history

The pre-publication history for this paper can be accessed here:



## Figures and Tables

**Table 1:** Patient characteristics and unadjusted medical health care utilization of patients with diabetes specified for absence/presence of comorbidity and type of comorbidity (% or mean (standard deviation (SD) (n = 7,499))

	**DM only**	**DM + any comorbidity (n = 3,324)**			**Type of comorbidity**			
					
					**DM + only dm-related comorbidity**	**DM + only non dm-related comorbidity**	**DM + both types of comorbidity**	
**Number of comorbidities**	**-**	**1**	**2**	**≥3**				
						
	**(n = 4,175)**	**(n = 2,419)**	**(n = 718)**	**(n = 187)**	**(n = 871)**	**(n = 1,856)**	**(n = 597)**	
	%/Mean (SD)	%/Mean (SD)	%/Mean (SD)	%/Mean (SD)	%/Mean (SD)	%/Mean (SD)	%/Mean (SD)	P-value
Percentage of total (%)	55.7	32.3	9.6	2.5	11.6	24.7	8.0	
								
**Patient characteristics**								
Sex (% male)	48.4	43.7	40.4	41.2	51.2	39.0	42.5	0.000
Mean age (in years)	62.6	66.9	71.1	72.9	71.5	64.9	73.1	0.000
Educational level (%) #								
Low	43.2	49.8	55.6	59.4	56.1	47.3	58.3	0.000
Middle	46.3	42.7	40.8	35.3	37.3	45.4	37.7	
High	10.5	7.4	3.6	5.3	6.5	7.3	4.0	
Mean number of comorbidities					1.11	1.13	2.34	0.000
								
**Medical health care utilization**								
*GP care*								
% of patients with GP contacts	100	100	100	100	100	100	100	
Number of contacts with GP (per year)	8.9 (6.5)	13.7 (8.60)	20.8 (12.8)	28.8 (15.9)	15.0 (10.6)	14.3 (9.1)	23.2 (14.0)	0.000
% of patients with prescriptions	97.0	98.5	99.3	98.4	99.4	98.2	99.2	0.000
Number of prescriptions (per year)	18.1(16.8)	25.9 (21.0)	39.8 (31.3)	57.1 (44.2)	32.0 (25.3)	25.6 (23.0)	44.4 (33.9)	0.000
								
*Ambulatory medical specialist care*†								
% of patients with consultations	12.3	23.0	30.4	38.0	17.8	26.5	33.3	0.000
Number of consultations (per year)	2.1 (1.4)	2.7 (1.7)	3.4 (2.0)	4.0 (2.3)	2.7 (1.6)	2.8 (1.8)	3.6 (2.2)	0.000
								
*Hospital care*								
% of patients with hospital admission†	5.1	14.7	23.1	31.0	13.8	16.2	26.5	0.000
Number of hospital admissions (per year)	1.7 (1.3)	2.3 (2.1)	2.9 (2.8)	3.2 (2.3)	2.7 (2.3)	2.3 (2.3)	2.9 (2.5)	0.000
Average length of stay (per admission)	6.7 (10.6)	6.3 (7.7)	8.0 (7.5)	11.2 (11.4)	7.9 (7.8)	5.8 (7.6)	9.5 (9.1)	0.001
Total hospital days (per year)	10.7 (16.3)	14.8 (21.0)	22.4 (26.5)	31.9 (27.3)	21.1 (24.4)	14.2 (22.1)	25.3 (25.6)	0.000

DM: diabetes mellitus; GP: general practitioner. # low: none and primary school; middle: secondary school or lower and intermediate vocational training; high: higher vocational training or university. †= only first referrals are included in the analysis.

**Table 2:** Effect of comorbidity of patients with diabetes on medical health care utilization adjusted for age, gender, educational level and GP practice specified for type of comorbidity with the patient group without comorbidity as reference group (coefficient and 95% confidence interval (CI) and Odds Ratio (OR) and 95% CI (n = 7,499))

	**DM + any comorbidity (n = 3,324)**						**Type of comorbidity**					
							
							**DM + only dm-related comorbidity**		**DM + only non dm-related comorbidity**		**DM + both types of comorbidity**	
**Number of comorbidities**	**1 (n = 2,419)**		**2 (n = 718)**		**≥3 (n = 187)**		**(n = 871)**		**(n = 1,856)**		**(n = 597)**	
							
	β/OR	CI 95%	β/OR	CI 95%	β/OR	CI 95%	β/OR	CI 95%	β/OR	CI 95%	β/OR	CI 95%
*GP care*												
No. of visits to GP (β)	3.9	(3.6, 4.3)	10.3	(9.7, 10.9)	18.0	(16.9, 19.1)	4.6	(4.1, 5.2)	4.7	(4.2, 5.1)	12.4	(11.7, 13.0)
% patients with prescriptions (OR)	*		*		*		*		*		*	
No. of prescriptions (β)	6.9	(5.9, 7.9)	19.2	(17.6, 20.8)	35.9	(33.0, 38.7)	11.7	(10.3, 13.2)	7.0	(5.9, 8.0)	23.4	(21.7, 25.2)
												
*Ambulatory medical specialist care*†												
% of patients with consultations (OR)	2.4	(2.1, 2.8)	3.7	(3.1, 4.6)	5.6	(4.0, 7.9)	1.7	(1.4, 2.1)	2.9	(2.5, 3.3)	4.3	(3.5, 5.4)
No. of consultations (β)	0.4	(0.3, 0.4)	0.8	(0.7,0.9)	1.3	(1.1, 1.5)	0.2	(0.1, 0.3)	0.5	(0.4, 0.6)	1.0	(0.8, 1.1)
												
*Hospital care*												
% patients with hospital admission (OR)	3.4	(2.9, 4.2)	6.4	(5.1, 8.2)	9.8	(6.8, 14.0)	3.3	(2.5, 4.2)	3.8	(3.2, 4.6)	7.7	(6.0, 9.9)
												
No. of hospital admissions (β)	0.3	(0.2, 0.3)	0.6	(0.5, 0.7)	0.9	(0.8, 1.1)	0.3	(0.2, 0.4)	0.3	(0.2, 0.4)	0.7	(0.6, 0.8)
Average length of stay (β)	0.6	(0.4, 0.7)	1.4	(1.1, 1.7)	3.0	(2.5, 3.6)	0.7	(0.4, 0.9)	0.6	(0.4, 0.9)	2.1	(1.8, 2.4)
Total hospital days (β)	1.5	(1.1, 2.0)	4.5	(3.7, 5.2)	9.1	(7.8, 10.4)	2.2	(1.5, 2.8)	1.7	(1.2, 2.2)	5.9	(5.1, 6.7)

DM: diabetes mellitus; GP: general practitioner. # low: none and primary school; middle: secondary school or lower and intermediate vocational training; high: higher vocational training or university. †= only first referrals are included in the analysis.

**Table 3:** Effect of comorbidity of patients with diabetes on medical health care utilization adjusted for age, gender, educational level and GP practice specified by comorbidity with the patient group without comorbidity as reference group (coefficient and 95% confidence interval (CI) and Odds Ratio (OR) and 95% CI)

**DM-related comorbidity**	**Heart diseases (n = 1,096)**		**Stroke (n = 218)**		**Retinopathy (n = 63)**		**Nephropathy (n = 57)**		**Diabetic Foot (n = 204)**	
	β/OR	CI 95%	β/OR	CI 95%	β/OR	CI 95%	β/OR	CI 95%	β/OR	CI 95%
*GP care*										
No. of visits to GP (β)	5.2	(4.74, 5.73)	3.4	(2.4, 4.4)	3.20	(1.37, 5.04)	5.6	(3.7, 7.6)	8.46	(7.42, 9.48)
% patients with prescriptions (OR)	*		*		*		*		*	
No. of prescriptions (β)	12.8	(11.52, 14.1)	4.1	(1.6, 6.7)	5.62	(0.90, 10.36)	12.1	(7.2, 17.0)	20.88	(18.23, 23.54)
										
*Ambulatory medical specialist care*†										
% of patients with consultations (OR)	1.7	(1.5, 2.1)	2.0	(1.5, 2.9)	1.4	(0.8, 2.7)	1.0	(0.5, 1.9)	1.0	(0.7, 1.5)
No. of consultations (β)	0.4	(0.3, 0.4)	0.4	(0.2, 0.6)	-0.2	(-0.1, 0.5)	-0.1	(-0.5, 0.2)	0.0	(-0.2, 0.2)
										
*Hospital care*										
% patients with admission (OR)	2.6	(2.2, 3.2)	2.8	(2.0, 4.1)	1.9	(1.00, 3.7)	0.9	(0.4, 1.9)	1.4	(0.9, 2.1)
No. of hospital admissions (β)	0.4	(0.3, 0.4)	0.4	(0.2, 0.5)	0.3	(0.1, 0.6)	0.2	(-0.1, 0.4)	0.1	(-0.0, 0.2)
Average length of stay (β)	0.9	(0.7, 1.1)	1.5	(1.0, 2.0)	0.7	(-0.2, 1.6)	-0.2	(-1.1,0.7)	0.8	(0.3, 1.3)
Total hospital days (β)	3.1	(2.5, 3.7)	3.3	(2.1, 4.4)	2.1	(0.0, 4.3)	0.8	(-1.5, 3.1)	2.4	(1.2, 3.6)

**Non DM-related comorbidity**	**Depression (n = 295)**		**Neurological diseases (n = 69)**		**Musculoskeletal Diseases (n = 1,722)**		**Lung Diseases (n = 531)**		**Cancer (n = 206)**	
	β/OR	CI 95%	β/OR	CI 95%	β/OR	CI 95%	β/OR	CI 95%	β/OR	CI 95%

*GP care*										
No. of visits to GP (β)	6.8	(5.9, 7.6)	4.1	(2.4, 5.9)	4.1	(3.7, 4.5)	5.3	(4.6, 5.9)	5.9	(4.9, 6.9)
% patients with prescriptions (OR)	*		*		*		*		*	
No. of prescriptions (β)	12.9	(10.6, 15.1)	8.0	(3.5, 12.5)	4.2	(3.2, 5.3)	15.3	(13.6, 17.0)	3.4	(0.8, 6.1)
										
*Ambulatory medical specialist care*†										
% of patients with consultations (OR)	1.1	(0.8, 1.5)	1.0	(0.5, 1.8)	3.6	(3.1, 4.1)	1.2	(1.0, 1.5)	1.3	(0.9, 1.9)
No. of consultation by specialist (β)	0.2	(0.1, 0.3)	0.1	(-0.2, 0.3)	0.6	(0.6, 0.7)	0.3	(0.2, 0.4)	0.4	(0.2, 0.5)
										
*Hospital care*										
% patients with admission (OR)	1.3	(0.9, 1.9)	1.5	(0.8, 2.9)	3.8	(3.2, 4.4)	1.2	(0.9, 1.6)	1.8	(1.2, 2.7)
No. of hospital admissions (β)	0.1	(0.0, 0.2)	0.0	(-0.3, 0.2)	0.3	(0.3, 0.4)	0.1	(0.1, 0.2)	0.4	(0.2, 0.5)
Average length of stay (β)	0.5	(0.1, 0.9)	0.5	(-0.3, 1.4)	0.9	(0.7, 1.1)	0.4	(0.0, 0.7)	0.5	(0.0,1.0)
Total hospital days (β)	1.5	(0.5, 2.6)	0.1	(-1.9, 2.2)	2.3	(1.8, 2.7)	1.47	(0.7, 2.2)	2.4	(1.2, 3.6)

DM: diabetes mellitus; GP: general practitioner *= ORs for getting pharmaceuticals prescribed can not be calculated due to the high percentages of patients who get pharmaceuticals prescribed; † = only first referrals are included in the analysis.

## References

[B1] Feinstein A (1967). Clinical judgement.

[B2] Beckman JA, Creager MA, Libby P (2002). Diabetes and atherosclerosis: epidemiology, pathophysiology, and management. JAMA.

[B3] Adler AI, Stratton IM, Neil HAW, Yudkin JS, Matthews DR, Cull CA, Wright AD, Turner RC, Holman RR, on behalf of the UK Prospective Diabetes Study Group (2000). Association of systolic blood pressure with macrovascular and microvascular complications of type 2 diabetes (UKPDS 36): prospective observational study. BMJ.

[B4] Anderson RJ, Freedland KE, Clouse RE, Lustman PJ (2001). The prevalence of comorbid depression in adults with diabetes: a meta-analysis. Diabetes Care.

[B5] Egede LE, Zheng D, Simpson K (2002). Comorbid depression is associated with increased health care use and expenditures in individuals with diabetes. Diabetes Care.

[B6] Van den Akker M, Buntinx F, Metsemakers JF, Roos S, Knottnerus JA (1998). Multimorbidity in general practice: prevalence, incidence, and determinants of co-occurring chronic and recurrent diseases. J Clin Epidemiol.

[B7] Black SA (1999). Increased health burden associated with comorbid depression in older diabetic Mexican Americans. Results from the Hispanic Established Population for the Epidemiologic Study of the Elderly survey. Diabetes Care.

[B8] Gijsen R, Hoeymans N, Schellevis FG, Ruwaard D, Satariano WA, van den Bos GAM (2001). Causes and consequences of comorbidity: a review. J Clin Epidemiol.

[B9] Westert GP, Satariano WA, Schellevis FG, van den Bos GAM (2001). Patterns of comorbidity and the use of health services in the Dutch population. European Journal of Public Health.

[B10] Norlund A, Apelqvist J, Bitzen PO, Nyberg P, Schersten B (2001). Cost of illness of adult diabetes mellitus underestimated if comorbidity is not considered. J Intern Med.

[B11] Carral F, Aguilar M, Olveira G, Mangas A, Domenech I, Torres I (2003). Increased hospital expenditures in diabetic patients hospitalized for cardiovascular diseases. J Diabetes Complications.

[B12] Rapoport J, Jacobs P, Bell NR, Klarenbach S (2004). Refining the measurement of the economic burden of chronic diseases in Canada. Chronic Dis Can.

[B13] Brandle M, Zhou H, Smith BR, Marriott D, Burke R, Tabaei BP, Brown MB, Herman WH (2003). The direct medical cost of type 2 diabetes. Diabetes Care.

[B14] O'Brien JA, Shomphe LA, Kavanagh PL, Raggio G, Caro JJ (1998). Direct medical costs of complications resulting from type 2 diabetes in the U.S. Diabetes Care.

[B15] Simpson SH, Corabian P, Jacobs P, Johnson JA (2003). The cost of major comorbidity in people with diabetes mellitus. CMAJ.

[B16] Westert GP, Schellevis FG, De Bakker DH, Groenewegen PP, Bensing JM, van der ZJ (2005). Monitoring health inequalities through general practice: the Second Dutch National Survey of General Practice. Eur J Public Health.

[B17] Lamberts H, woods M, Hofmans-Okkes I (1993). The international classification of primary care in the European Community.

[B18] Zorginformatie SIG (1999). Userguide National Ambulant Register, version 3.0/PHD (in Dutch) Gebruikershandleiding Landelijke ambulante zorgregistratie, versie 3.0/PHD.

[B19] Paas GRA, Veenhuizen KCW (2002). Research on the reliability of the National Ambulent Register (in Dutch). Onderzoek naar de betrouwbaarheid van de LMR.

[B20] Registratie SM (1980). SMR: 1980: Classification of Diseases, 1980 based on the International Classification of Diseases 9th revision (In Dutch). Classificatie van ziekten 1980, Gebaseerd op de internationale classificatie van ziekten, Clinical Modification (ICD9-CM), 1980.

[B21] Reitsma JB, Bonsel GJ, Heisterkamp SH (1999). Registers in cardiovascular Epidemiology. Registers in Cardiovascular Epidemiology.

[B22] Struijs JN, Baan CA, Hutten JBF, Westert GP (2003). Possibilities of linking data on referrals by general practitioners with data on hospital utilization: a pilot study (in Dutch). De mogelijkheden van koppeling van geanominiseerde huisarts- en ziekenhuisgegevens. Een vooronderonderzoek. Tijdschrift voor Gezondheidswetenschappen.

[B23] van Weel C, Schellevis FG (2006). Comorbidity and guidelines: conflicting interests. Lancet.

[B24] Monane M, Kanter DS, Glynn RJ, Avorn J (1996). Variability in length of hospitalization for stroke. The role of managed care in an elderly population. Arch Neurol.

[B25] Hanefeld M, Fischer S, Schmechel H, Rothe G, Schulze J, Dude H, Schwanebeck U, Julius U (1991). Diabetes Intervention Study. Multi-intervention trial in newly diagnosed NIDDM. Diabetes Care.

[B26] de Bruin A, de Bruin EI, Bestand PGS (2003). Linking data of National Ambulant Register and GBA data: methods, results and quality research (in Dutch). Koppeling van LMR- en GBA gegevens: methode, resultaten en kwaliteitsonderzoek.

[B27] Stratton IM, Kohner EM, Aldington SJ, Turner RC, Holman RR, Manley SE, Matthews DR (2001). UKPDS 50: risk factors for incidence and progression of retinopathy in Type II diabetes over 6 years from diagnosis. Diabetologia.

[B28] Rupp I, Boshuizen HC, Jacobi CE, Dinant HJ, van den BG (2004). Comorbidity in patients with rheumatoid arthritis: effect on health-related quality of life. J Rheumatol.

[B29] Battersby MW (2005). Health reform through coordinated care: SA HealthPlus. BMJ.

